# Pathogenetical and Neurophysiological Features of Patients with Autism Spectrum Disorder: Phenomena and Diagnoses

**DOI:** 10.3390/jcm8101588

**Published:** 2019-10-02

**Authors:** Yunho Jin, Jeonghyun Choi, Seunghoon Lee, Jong Won Kim, Yonggeun Hong

**Affiliations:** 1 Department of Rehabilitation Science, Graduate School of Inje University, Gimhae 50834, Korea; jynh33@naver.com (Y.J.); yiopiop0011@nate.com (J.C.); 2 Ubiquitous Healthcare & Anti-aging Research Center (u-HARC), Inje University, Gimhae 50834, Korea; 3 Biohealth Products Research Center (BPRC), Inje University, Gimhae 50834, Korea; 4 Department of Physical Therapy, College of Healthcare Medical Science & Engineering, Inje University, Gimhae 50834, Korea; 5 Gimhae Industry Promotion & Biomedical Foundation, Gimhae 50969, Korea; 6 Department of Healthcare Information Technology, College of Bio-Nano Information Technology, Inje University, Gimhae 50834, Korea; 7 Department of Medicine, Division of Hematology/Oncology, Harvard Medical School-Beth Israel Deaconess Medical Center, Boston, MA 02215, USA

**Keywords:** autism spectrum disorder, melatonin, neuropsychiatric disorder, neuroinflammation, electroencephalography, event-related potential

## Abstract

Autism spectrum disorder (ASD) is a neurodevelopmental disorder that is accompanied by social deficits, repetitive and restricted interests, and altered brain development. The majority of ASD patients suffer not only from ASD itself but also from its neuropsychiatric comorbidities. Alterations in brain structure, synaptic development, and misregulation of neuroinflammation are considered risk factors for ASD and neuropsychiatric comorbidities. Electroencephalography has been developed to quantitatively explore effects of these neuronal changes of the brain in ASD. The pineal neurohormone melatonin is able to contribute to neural development. Also, this hormone has an inflammation-regulatory role and acts as a circadian key regulator to normalize sleep. These functions of melatonin may play crucial roles in the alleviation of ASD and its neuropsychiatric comorbidities. In this context, this article focuses on the presumable role of melatonin and suggests that this hormone could be a therapeutic agent for ASD and its related neuropsychiatric disorders.

## 1. Introduction

Autism spectrum disorder (ASD) is defined as a neurodevelopmental disorder characterized by social deficits, repetitive and restricted interests, and altered development of the brain [1]. The prevalence of ASD in children and adolescents is approximately 0.6%–1% [2] The proportion of the adult population afflicted with ASD is estimated to be 2.4%–9.9% [3]. However, a large number of patients not only suffer from ASD but also have other neuropsychiatric conditions. This pattern of neuropsychiatric comorbidity was first suggested in 1978 [4]. More recently, nearly three-fourths of the patients with ASD were revealed to have neuropsychiatric conditions in addition to their core symptoms [5]. It has been documented that maternal inflammation may increase the risk of ASD [6]. Also, neuroinflammation may affect fetal brain development [7]. Indeed, neuroinflammatory cascades have been proved to be accompanied by autism in the brain [8]. In turn, it has widely been reported that structural abnormalities in the brain may be linked to neuropsychiatric problems [9,10]. There is an accumulation of literature on the use of electroencephalography (EEG) to objectively measure those neuronal changes in ASD [11,12,13]. Additionally, the unusual structural development in the brain may occur with misregulation of neuroinflammation in patients with congenital neurodevelopmental problems, increasing the risk of neuropsychiatric disorders. However, the proper intervention for patients with ASD and its neuropsychiatric comorbidities has not been fully established. Since the majority of ASD patients suffer not only from ASD itself but also its neuropsychiatric comorbidities, therapeutic agents for ASD must be studied in multiple directions.

Numerous researchers have focused on ASD, but ASD treatment still remains challenging, as the etiology of ASD is poorly understood. ASD treatment tends to be applied mainly on the basis of symptoms, and the lack of a specific etiologic diagnosis makes it difficult to develop a fundamental therapeutic approach to ASD. Currently, risperidone and aripiprazole, which are antipsychotic drugs, are approved by the US Food and Drug Administration (FDA) for ASD symptom treatment [14]. However, the use of risperidone has some side effects, including appetite increase, drowsiness, and an increase in prolactin [14,15]. Similarly, sedation, fatigue, vomiting, and an increase in appetite are the side effects of aripiprazole use [14,16]. Recently, the pineal neurohormone melatonin has been suggested as a novel interventional candidate, especially for the ASD patients codiagnosed with Fragile X syndrome (FXS), indicating that melatonin may be involved in correcting disrupted circadian rhythm found in those patients [17]. In addition to FXS, other known syndromic ASDs were proven to be alleviated by melatonin. Similarly, sleep disturbance in Rett syndrome patients appears to be relieved by this hormone, making it useful for those with Rett syndrome [18]. This hormone has also been used for the treatment of sleep disorders in tuberous sclerosis [19]. As melatonin is naturally present in the body of living creatures, this hormone is expected to have few or no side effects when administered. Also, a large number of studies have suggested that melatonin is able to modulate neuroinflammation [20,21]. In other words, melatonin is expected to alleviate the neuroinflammation associated with ASD and to ultimately be therapeutically efficacious in the treatment of structural brain abnormalities and neuropsychiatric disorders. In this regard, this review article suggests that melatonin is a promising therapeutic agent for patients with ASD.

## 2. The Importance of Synaptic Pruning by Autophagy in ASD Pathophysiology

**Figure 1:**
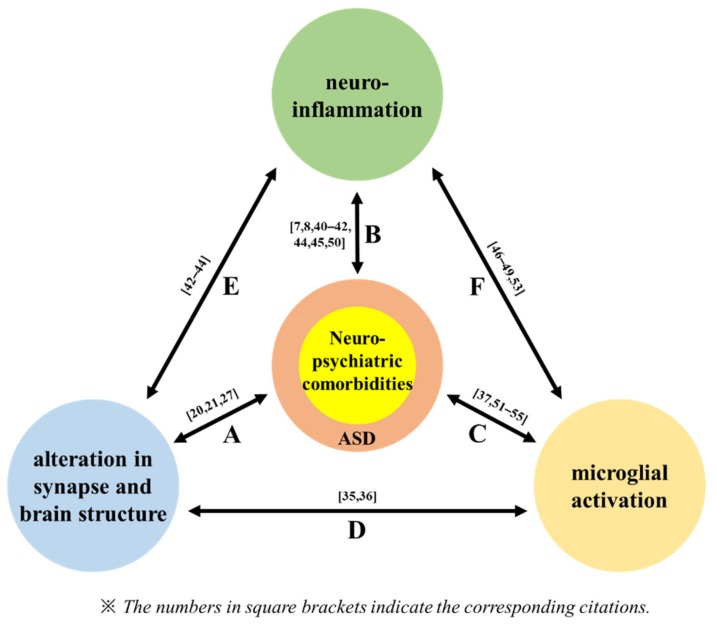
Most autism spectrum disorder (ASD) patients have neuropsychiatric comorbidities caused by neuroinflammation and structural alterations in the brain. Also, microglial activation influences both brain structure and inflammation. Therefore, these factors interact in terms of ASD pathophysiology and the comorbid neuropsychiatric conditions, and therapeutic strategies for ASD should focus on these factors. The numbers in square brackets indicate the reference numbers of the cited studies. (**A**) In an ASD brain, synaptic pruning is abnormally decreased, followed by increased synaptic spine density. (**B**) Increased concentrations of inflammatory cytokines in ASD brains. (**C**) A dense population of microglia in an ASD brain. (**D**) Synaptic connectivity is maintained via microglia-mediated synaptic pruning, followed by removal of inappropriate synapses. (**E**) Structural changes in brains that are in a pro-inflammatory state. (**F**) Microglia play inflammatory roles in terms of the degradation of toxic agents and inflammatory cytokine release, and also serve as major immunoeffectors.

Various studies have reported that several factors are associated with the emergence of ASD. Among them, altered dendritic spine density is thought to be related to ASD. In a previous study, ASD brains showed an increased average spine density (24.60 per 25 μm) compared with that of age-matched controls (20.47 per 25 μm) [22]. Similarly, it has been reported that altered synaptic structures can be seen in diverse ASD animal models [23]. In this context, abnormal synaptic structure is thought to be closely associated with the development of ASD. During brain maturation, synaptic pruning is generally induced [24,25]. Therefore, this process is crucial for normal brain development [26]. Postnatal synaptic development in the cerebral cortex involves simultaneous synaptic formation and pruning [27,28,29]. Synaptic formation is more prevalent than pruning in early childhood, while synaptic pruning predominates formation from late childhood to adulthood [29,30]. However, synaptic pruning in an ASD brain decreases abnormally, followed by a high synaptic spine density (Figure 1A) [29]. Notably, adolescent ASD patients exhibited a 40% increase in synaptic spine density within the pyramidal neuron V layer, and childhood ASD patients an 8% increase, compared to age-matched controls, implying that ASD is associated with defective synaptic pruning. Numerous researchers have insisted that autophagy machinery plays a role in ASD pathogenesis. The term autophagy, which literally means self-eating, is a process that degrades damaged proteins to recycle them into a new energy source for new protein synthesis, leading to the recovery of cell homeostasis [31]. Damaged or long-lived proteins are sequestered into the autophagosome—a double-membrane cytosolic vesicle—which then fuses with the lysosome [31,32]. Then, the target protein undergoes lysosomal degradation and becomes a new energy source for the synthesis of new proteins [31,33]. Autophagy is thought to contribute to synaptic homeostasis [34]. Specifically, autophagy is required for spine pruning and the subsequent correction of pathologies and social behavior deficits of ASD [29]. In an ASD brain, synaptic deficit occurs due to impaired autophagy following the hyperactivation of mammalian target of rapamycin (mTOR), which is an autophagy inhibitory regulator [29,35]. Indeed, mTOR disinhibition in mice impaired dendritic arborization as evidenced by a 24.9% reduction in dendritic spine density within the molecular layer, and autistic behaviors, implying that the autophagic machinery does not function appropriately in ASD patients [36].

**Figure 2:**
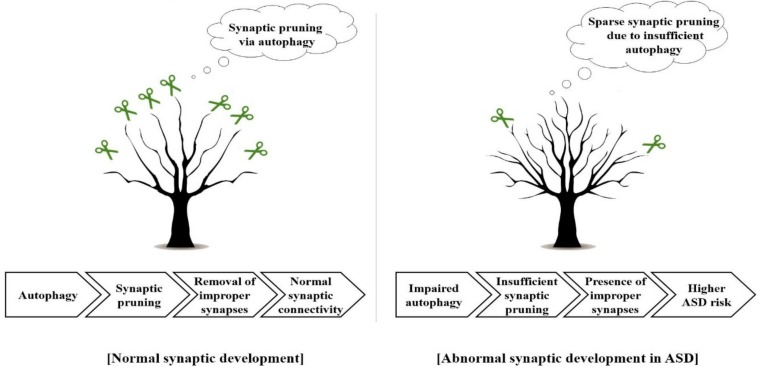
ASD risk is reduced when synaptic pruning is achieved through autophagy. Autophagy marked as scissors removes improper synapses, leading to appropriate synaptic pruning and, ultimately, normal synaptic connectivity which expressed as straight branches. However, abnormal synaptic connectivity due to impaired autophagy and resultant insufficient synaptic pruning may increase the risk of ASD.

Moreover, around a two-fold higher phosphor-mTOR/total-mTOR ratio was found in the temporal lobe of ASD patients aged 13–20 compared with that of age-matched controls [29]. Therefore, proper synaptic pruning is achieved if autophagy is appropriately activated via the optimal activation of mTOR. In turn, alleviating the synaptic pathology may contribute to the amelioration of ASD. Proper autophagy, which is required for synaptic connectivity, is closely related to microglia function. Postnatally, normal synaptic connectivity is maintained via microglia-mediated synaptic pruning, followed by removal of inappropriate synapses (Figure 1D) [37,38]. Disruption of autophagy may reduce synaptic pruning, subsequently increasing improper synapses [39]. Indeed, mice with Atg7-deficient microglia showed ASD-like behaviors, including social deficits and repetitive behaviors [39]. Furthermore, positron emission tomography (PET) has revealed that multiple ASD brain areas, including the cerebellum, midbrain, pons, fusiform gyri, cingulate cortex, and orbitofrontal cortex, exhibit microglial activation [40]. Similarly, such activation was observed in ASD-like animal models, indicating that microglia play roles in synaptic pruning [41]. In this regard, autophagy in microglia appears to play a role in normal synaptic development and is expected to reduce the risk of ASD phenotype emergence (Figure 2).

## 3. Regulation of Neuroinflammation Might Be Required for the Alleviation of ASD

The ASD brain was proven to have a consistently and significantly higher concentration of inflammatory cytokines compared with brain tissue of a control [8]. According to a previous study where elevated pro-inflammatory cytokines were found, there was an increase not only in macrophage chemoattractant protein-1 (MCP-1) but also in thymus and activation-regulated chemokine (TARK) in the anterior cingulate gyrus (2.2- and 2.63-fold increases, respectively) and cerebellar hemisphere (1.9- and 1.09-fold increases, respectively) of ASD brains [8]. Likewise, inflammatory cytokines were abundant in the cerebrospinal fluid (CSF) of ASD patients: MCP-1, interleukin-6 (IL-6), interferon-γ (IFN-γ), interleukin-8 (IL-8), macrophage inflammatory protein-1β (MIP-1β), neutrophil activating peptide-2 (NAP-2), interferon-γ inducing protein-10 (IP-10), and angiogenin were increased in the CSF of ASD patients compared with the levels of these proteins in controls (Figure 1B) [8,42]. As these cytokines are crucial for inflammatory responses, the increased concentrations of pro-inflammatory cytokines imply that inflammatory responses following ASD may be occurring. Similar to this result, enhanced anti-inflammatory cytokine IL-10 levels during the prenatal stage were found to be favorable to cognitive and behavioral development [43]. Taken together, the balance between pro- and anti-inflammatory cytokines is fundamental for normal fetal brain development [7].

These increased neuroinflammatory cytokines appear to be associated with brain structure. Indeed, structural changes were found in brains that were in a pro-inflammatory state [44]. Also, reduced hippocampal volume and increased inflammatory cytokine levels, including IL-6 and C-reactive protein (CRP), were observed in patients with major depressive disorder (Figure 1E) [45]. Moreover, experimental models of ASD revealed reductions in the diameter and thickness within hippocampal dentate gyrus cells (16% and 19% reductions on day 14 post-induction, respectively), compared to control animals [46].

Neuroinflammation seems to affect both neuropsychiatric symptoms and brain structure. Subjects with increased blood CRP levels not only suffered from depression but also evidenced altered hippocampal structures, including 11% reductions in the CA1 area and the thickness of the subiculum [47]. Similarly, increased levels of inflammatory cytokines, such as tumor necrosis factor-α (TNF-α), IL-1, and IL-6, can diminish positive mood and induce anxiety [44]. Furthermore, the enhancement of pro-inflammatory cytokines led to a depressive mood in healthy subjects [48]. As mentioned in Section 2 above, microglia affect synaptic development. In addition to their role in synaptic connectivity, microglia also have inflammatory roles, such as degrading toxic agents, releasing inflammatory cytokines, and functioning as principal immunoeffectors (Figure 1F) [49,50,51]. That is to say, inflammation might be a causative factor in ASD phenotypes [52]. As ASD has been reported to be associated with brain inflammation [53], ASD patients have shown an increased number of microglia in their brains compared with that in the brains of healthy controls [54]. Additionally, the population of microglial cells in the fronto-insular and visual cortex of ASD brains was denser than that of control brains (Figure 1C) [55]. Likewise, induction of inflammatory mediators in pregnant mice caused not only an increase in the microglial population but also ASD phenotypes in the offspring [56]. Also, a broad range of microglial activations was observed by PET measurement in the cerebellum, brainstem, anterior cingulate cortex, frontal cortex, temporal cortex, parietal cortex, and corpus callosum of young ASD brains [40]. These results indicate that ASD is closely connected to inflammation. Presumably, prenatal inflammation might have occurred. Neuropsychiatric disorders like ASD apparently accompany microglial activation [57]. Typically, microglial activation is thought to be the major reason why proinflammatory cytokine levels rise after brain damage [58]. This pathology can be improved by melatonin; the hormone inhibits microglial activation and ameliorates neuroinflammation-induced neural degeneration [59]. Accordingly, regulation of inflammation via melatonin-mediated reduction of microglial activation may alleviate ASD.

## 4. Electrophysiology in ASD

**Figure 3:**
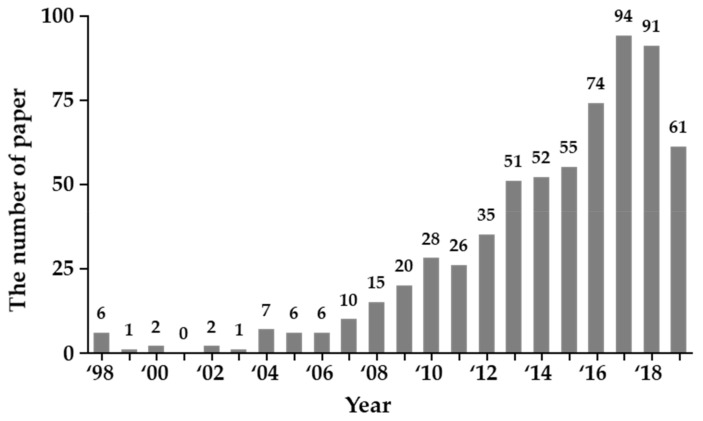
The number of papers published on the topic of ASD with either electroencephalography (EEG) or event-related potential (ERP) analysis. Data source is from PubMed as of June 2019. Note that data in the year 1998 includes all publications before and in 1998.

EEG has been widely used to investigate electrophysiological changes of the brain in patients with mental and psychiatric disorders, such as epilepsy, attention deficit hyperactivity disorder (ADHD), depression, and ASD [60,61,62,63,64]. EEG is designed to indirectly measure neuronal activities in the brain when (i) a patient is resting or (ii) he/she is responding to external stimuli (e.g., sound, light, images of faces). The latter is to elicit an event-related potential (ERP) of the brain associated with specific tasks [65,66,67]. There are many advantages of using EEG in the study of ASD. For example, EEG is a non-invasive measure, has a higher temporal resolution (at the cost of relatively poor spatial resolution though), and could be applied more widely with higher tolerance for movements, compared to other techniques such as magnetic resonance imaging (MRI). The number of publications on applying EEG/ERP techniques on the study of ASD is growing rapidly, as seen in Figure 3. In this chapter, we provide a brief overview of analyses on resting EEG and ERP in recent ASD researches.

### 4.1. Detection of Paroxysmal or Epileptic Forms in Resting EEG

The epileptic waveform and abnormal paroxysmal activity have been reported to occur frequently in ASD [68,69]. The abnormal EEG wave form could be characterized as spikes (i.e., sharp waves having pointed peaks) that last circa 50 milli-seconds (ms). The detection of the abnormality would require EEG recordings in a relatively long period and sometimes the detection would be viable only when patients are asleep [60,70]. In ASD, the abnormal activities occur in “isolated” manners (i.e., without identifiable seizures), and should be differentiated from inter-ictal spike discharges that occur between seizures [64]. Despite the high comorbidity of epileptic activity with ASD, there is no clear association between the abnormalities and the symptoms of ASD. Also, it is not clearly known if such EEG abnormalities in children with ASD are likely to develop into a seizure. Since the detection of the waveforms is mostly based on visual inspections of EEG signals by experts, the interrater reliability would also contribute to the large variability in the recognized prevalence of EEG abnormalities. Despite the fact that a large portion of ASD patients, either with or without epilepsy, have EEG abnormalities, there are not enough evidences for the use of EEG analysis to screen ASD or to monitor responses to treatments [71,72]. Recently, there are many studies to identify epileptic waveforms via computer-aided methods, including wavelets and deep learning [73,74,75,76]. These new advancements in the area of biomedical engineering would enable us to analyze larger ASD data sets and may help standardize diagnostic EEG measures for ASD in the future.

### 4.2. Spatio-Temporal Characteristics of Resting EEG in ASD

Resting EEG reflects electrophysiological states of the brain when patients make no response to any stimulus. This technique is thus relevant for young children who have difficulties in performing tasks accurately due to cognitive and/or perceiving impairments (e.g., ASD). In these regards, quantitative analysis of resting EEG could be useful for investigating the abnormal developments of ASD children [63,77,78,79,80,81]. It is also worthy of noting that resting EEG has been performed to monitor effects of neurofeedback training on ASD children [82,83,84]. The waveforms of resting EEG typically consist of “blunt”, random-like oscillations in various frequency ranges, in contrast to the “sharp” epileptic waveforms. A common way to characterize resting EEG is to decompose the waves into several rhythmic oscillations and calculate the strength of rhythms in given frequency bands (i.e., EEG absolute spectral power) via computational methods (e.g., Fourier transformation). A typical set of these bands is named as delta (1 to 3 Hz), theta (4 to 7 Hz), alpha (8 to 12 Hz), beta (13 to 30 Hz), and gamma (>30 Hz). In many quantitative EEG studies, relative spectral power, the amount of EEG activity in a given frequency band divided by the total amount of activity, is explored as well. There are increasing volumes of evidence that the rhythmic activities in those bands may reflect a broad variety of cognitive and sensorimotor processes [85,86]. Although some other advanced nonlinear techniques, such as wavelets, detrended fluctuation, and fractal dimension analyses, are also developed, the reliability of these techniques in studies of clinical EEGs is still arguable [87,88,89]. We thus mainly focused on spectral analysis in this short review. Some spatial properties, such as regional asymmetry and/or coherence among EEGs recorded at different placements will be discussed later in this subsection. With an extensive literature review, Wang et al. [63] reported a relatively consistent electrophysiological abnormality pattern in ASD. Specifically, they found a unique U-shaped profile of EEG power changes in ASD children at all stages of development [63]. That is, spectral power at delta/theta (low) and beta/gamma (high frequency) bands was enhanced, while power at alpha (middle frequency) band was reduced. Despite the general profile, the detailed outcomes of EEG abnormalities in ASD patients varied between studies, which is mainly due to confounding aspects of ASD (e.g., behavioral and putative heterogeneity). Also, rapid developments of EEG recording technology and quantitative analysis methods in recent decades may attribute the complexity in the outcomes of the resting EEG studies in ASD. In many ASD studies, EEGs are typically recorded at multiple placements, which enables us to investigate spatial features of the electrophysiological changes in ASD via comparing EEG characteristics at each location. A common method is to compare spectral power in the left and right hemispheres. In many recent papers, spectral power at all frequency bands in the left hemisphere are reported to be enhanced in ASD [63,90,91], which is very eminent compared to the enhancements generally seen in healthy adolescents [90]. Another common method is to compute coherence patterns among EEGs, which could yield a functional connectivity between the two regions of interest. In general, reduced long-range coherence patterns, especially around the frontal area, were reported recently [92,93], and are consistent with findings from functional MRI (fMRI) studies [94,95]. However, it should be note that not all studies uncover the same outcomes, which requires further examination on this topic with larger samples in order to associate the outcomes with possible traits of ASD. Some other advanced measures, such as (phase) synchrony, mutual information, and transfer entropy, were also applied to characterize EEGs in ASD. For example, Bosle et al. [96] suggested modified multiscale entropy as a potential biomarker for early detection of risk for ASD, while Thatcher et al. [97] demonstrated that instantaneous phase differences between two EEG recordings could be a biomarker of ASD. These findings necessitate further studies utilizing various resting EEG methods for the investigation of ASD.

### 4.3. Quantitative Analysis of ERP in ASD

Since deficits in responses to external stimuli are the core features of ASD, the ERP has been widely utilized to explore sensory processing in ASD in clinical settings from the early 1980s [65]. ERP is an electrophysiological activity induced by specific external stimuli (e.g., sound and light) within a short period (i.e., circa 500 ms). This electrical activity in the brain neuronal circuits exhibits specific temporal patterns across individuals consistently and is often embedded in a long period EEG recording. Typical ERP waveforms isolate specific response patterns to external stimuli and are distinctive from background noise. These could be obtained by averaging multiple EEG segments properly trimmed and aligned according to stimuli onsets. The resulting ERP typically contains several peaks often named as N1, P2, and so on. Here, the letters represent the direction of each peak (i.e., P for positive and N for negative) and the numbers denote the order of occurrence of peaks starting from a stimulus onset. The number is often replaced by a relative time difference between each peak and the onset. For example, P300 denotes that a positive ERP component occurs about 300 ms after a stimulus. The latency and amplitude of each peak are the two primary features of ERP, indicating the time needed for a stimulus to activate a brain region and the strength of collective neuronal activities in the region, respectively. Abnormality in these ERP features are often explored as biomarkers of sensory processing in ASD. A common finding on ERP is atypically increased latency of N100 in ASD [98], which may reflect functional changes of the connectivity of the neuronal networks in patients. Some other studies have also reported that the amplitudes of P50 and N100 are lower in ASD, which could reflect reduced feedback strengths in corticothalamic loops [99]. It should also be noted that ERP waveforms could be diverse depending on the modality of stimuli (e.g., auditory and/or visual) and experimental designs (e.g., novelty oddball paradigm). ERP could be further deviated when stimuli are associated with cognitive process (e.g., speech sound or familiar face) [100]. Use of these social ERP paradigms revealed the anomality of P300 amplitude and latency in ASD when stimuli were speech sounds [101]. Similarly, the anomality of N290 and P400 in ASD infants, and N170 in ASD adults were reported when they responded to faces [102]. In short, there exists a large number of evidences suggesting that neural responses evoked by sensory and/or social stimuli in ASD are different from those in healthy controls. However, it is still difficult to identify ERP as a consistent biomarker of ASD due to the lack of consistent findings between studies, which requests the standardization of experimental paradigms and the use of larger sample for ERP studies in ASD in the future.

## 5. Potential Therapeutic Intervention for ASD and Neuropsychiatric Comorbidities

### 5.1. Melatonin Contributes to Neural Development via Regulating Programmed Cell Death

During neural development, programmed cell death (PCD) plays a major role in shaping neuronal environment by regulating the numbers and types of cells in the central nervous system [50,103]. In particular, apoptosis is important in the regulation of neuronal cell death during neural development. A lack of apoptotic protease activating factor 1 (Apaf1), which initiates apoptosis [104], can cause abnormal brain enlargement, leading to deregulation of apoptosis and even prenatal death in experimental animals [105]. Other researchers have suggested that excessive apoptotic cell death attributed to a lack of the anti-apoptotic Bcl-x protein may cause neuronal immaturity in the developing brain and spinal cord, followed by embryonic death [106]. Several studies have shown that apoptotic cell death can be affected by melatonin. Pathophysiologically, neural injuries trigger excitotoxicity, dysregulation of calcium ion homeostasis, excessive nitric oxide synthesis, and apoptosis [107]. These changes may eventually cause apoptosis-mediated cell death and tissue damage, but such damage can be alleviated by melatonin-mediated neuroprotection [107] because melatonin can serve as a radical scavenger [108]. Melatonin seems to protect neuronal cells from apoptotic cell death-mediated neurotoxicity by regulating the expression of proteins associated with apoptotic cascades [109]. Notably, the elevated level of protection against apoptotic cell death is thought to be attributed to the melatonin-mediated enhancement of the interaction between pBad and 14-3-3 that results in the inhibited activation of apoptosis [110]. Following certain types of neural injury, cytochrome c is released from the mitochondria into the cytoplasm where, in turn, it activates the pro-apoptotic caspases 3, 6, and 9 by binding to Apaf1 and deoxyadenosine triphosphate (dATP) [111,112,113,114]. This pro-apoptotic process is thought to be blocked by melatonin, because melatonin maintains mitochondrial homeostasis [114]. Physiological clearance of unnecessary neuronal axons is carried out via apoptosis and synaptic pruning. In both pathways, neuronal degeneration occurs, but there are some differences: degeneration of whole neurons can be seen in apoptosis, while target axons are selectively degenerated in synaptic pruning [115]. Both apoptosis and synaptic pruning require the apoptotic protein caspase 3 and 6, implying that apoptotic proteins regulate synaptic pruning as well as apoptosis [115]. These apoptotic proteins are known to be regulated by melatonin [116]. This neurohormone suppresses apoptosis in normal cells, while it induces apoptosis in cancer cells [116]. Given that melatonin ameliorates ASD behaviors [117,118], it seems likely that it induces synaptic pruning rather than apoptosis, leading to an improvement in synaptic connectivity in ASD. Extensive studies are required to clarify the nature of synaptic pruning regulated by melatonin and apoptosis.

Another form of PCD, autophagy, has been reported to be closely related to melatonin and apoptosis. Luo et al. [119] suggested that melatonin enhances autophagy activation through the suppression of apoptosis. This melatonin-induced apoptosis-inhibiting effect on autophagy may play a beneficial role in the ASD brain. As described in the section above, inappropriate synapse removal and proper synaptic pruning for normal neural development are expected to occur through autophagy [39]. In the same context, many have insisted that autophagy activation during neurodevelopment is essential [120]. ASD risk in children was shown to be increased by a mutation in WDFY3 [121], which is a risk gene for developmental disabilities [122]. Autophagy in mitochondria enables mitochondrial quality control through WDFY3-mediated mitophagy [123], and, as a result, synaptic pruning and subsequent normal remodeling of neural circuits can be acquired. Consequently, autophagy as well as apoptosis, which is regulated by melatonin, is involved in neural development. Accordingly, these two PCD types presumably contribute to reduced ASD risk.

### 5.2. Melatonin-Mediated Promotion of Normal Sleep Ameliorates ASD by Facilitating Normal Neural Development

ASD is a complex disorder; the genetic influences in play are explained by genetic variations (Table 1) [124]. ASD is accompanied by altered gene expression in several brain regions. Specifically, the dorsolateral prefrontal cortex of the ASD brain exhibits reduced mGluR5 expression triggering inappropriate synaptic connectivity and abnormal brain functioning [41]. mGluR5-knockout animals exhibited ASD-like behaviors [41]. Similarly, the anterior cingulate gyrus, the motor cortex, and the thalamus of the ASD brain exhibited reduced levels of mitochondria-associated genes, including metaxin 2 (MTX2), the light polypeptide (NEFL), and solute carrier family 25 member 27 (SLC25A27), implying that ASD is associated with defective mitochondrial activity and energy metabolism [125]. Disruption of gene networks in ASD patients is also evidenced by high-level expression of ASD candidate genes in inhibitory neurons, indicating probable dysregulation of the inhibitory neuron transcriptome, in turn triggering an excitation/inhibition imbalance [126]. Similarly, neurodevelopment-related genes are dysregulated in the ASD brain [127]. Thus, because genetic factors influence ASD, the altered gene expression patterns of ASD can render brain growth abnormal [128].

**Figure 4:**
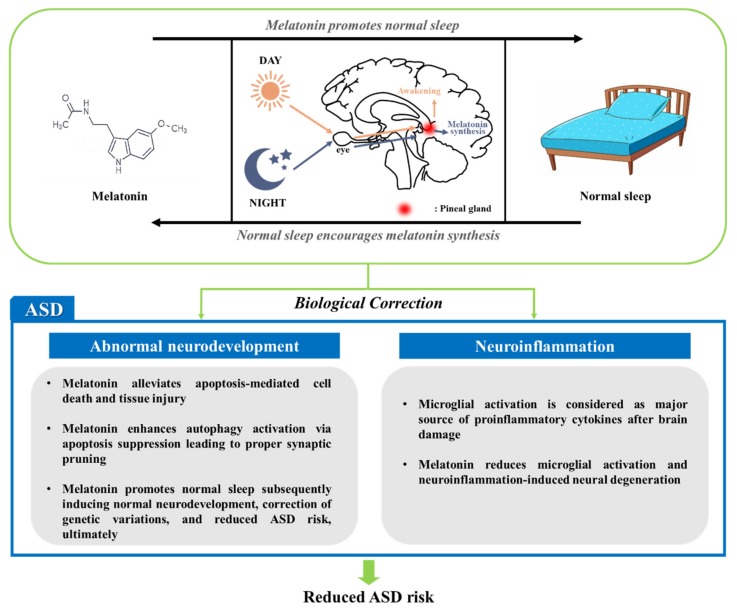
Beneficial effects of melatonin when the levels thereof are maintained by normal sleep. When a normal melatonin concentration is achieved, the hormone may correct abnormal neurodevelopment and neuroinflammation. Melatonin facilitates normal neurodevelopment not only by regulating programmed cell death (PCD) but also by promoting normal sleep. Also, melatonin reduces microglial activation, which greatly increases proinflammatory cytokine levels; therefore, melatonin inhibits neuroinflammation-induced neural degeneration.

When seeking to correct the abnormal neurodevelopment associated with ASD, normal sleep is recommended. Sleep is a vital physiological state that influences neural development [129]. Normal sleep is considered as a key factor for neurodevelopment [130]. Importantly, neuroplasticity of a developing brain can be achieved during sleep [131]. It has been unambiguously reported that neurodevelopmental/neuropsychiatric disorders, including ASD, are closely related to sleep and circadian rhythms [132,133]. Chronic disturbance of sleep causes disruption in the circadian clock in the brain [134]. In this context, ASD may be closely related to sleep disruption. During normal sleep, synthesis of proteins related to synaptic plasticity occurs [135]. These proteins are synthesized in a ratio mirroring the amount of rapid eye movement (REM) to non-REM sleep [136,137]. Sleep also regulates pro- and anti-inflammatory networks [138]. It has been documented that pro-inflammatory cytokines have sleep-regulating functions [137]. In particular, IL-1 and TNF-α are known to increase the duration of non-REM sleep [139]. As mentioned in Section 3, ASD patients have increased concentrations of pro-inflammatory cytokines. Namely, pro-inflammatory cytokines are thought to be elevated with ASD in order to regain neuronal homeostasis via regulation of sleep as well as inflammation. The sleep/wakefulness rhythm is regulated by the suprachiasmatic nucleus (SCN) in the hypothalamus [140]. SCN, which is known to highly express the melatonin receptor MT1 and MT2, has a neuronal activity-regulating function, and this function is controlled by melatonin [141,142,143]. Accordingly, melatonin is a key regulator of sleep/wakefulness. Also, melatonin has a sleep-promoting effect [142]. Indeed, melatonin improves total sleep time and decreases mental illnesses, indicating the close relationship between normal sleep and neuropsychiatric disorders [142,144,145]. Since melatonin plays crucial roles, appropriate sleep after normal melatonin secretion exerts a beneficial influence on ASD. The blood levels of melatonin and its metabolite 6-sulfatoxymelatonin are significantly lower in ASD patients [146]. This abnormal melatonin level is linked to autistic behaviors and thought to be attributable to genetic variations in melatonin pathways [147]. Maintenance of a normal melatonin level via normal sleep reduces the risk of ASD and its neuropsychiatric comorbidities not only by promoting normal neural development but also by controlling inflammation. Indeed, melatonin supplementation extends sleep duration, reduces awakenings, and triggers faster sleep onset, presumably reducing the risk of ASD by enhancing neurodevelopment. In this respect, the normalization of sleep would be important in the therapeutic strategy for treating ASD, and melatonin intervention may be an efficient means to achieve this. The relevant roles of melatonin in ASD and related comorbidities are summarized in Figure 4.

## 6. Conclusions

ASD is a neurodevelopmental and neuropsychiatric disorder from which many people suffer nowadays. However, the most effective treatment for ASD is still not known. This article suggests that some therapeutic potential may be found in melatonin for the treatment of ASD. Melatonin benefits neural development by regulating apoptosis, thus ASD, which is a kind of neurodevelopmental disorder, could be alleviated by melatonin treatment. Moreover, psychiatric comorbidities of ASD are thought to be ameliorated via melatonin treatment since this hormone not only regulates inflammation but also normalizes sleep. The regulation of inflammation and normal sleep are essential for neural development. Therefore, clinical application of melatonin may not only contribute to the regulation of inflammation but also to the entrainment of normal sleep, leading to reduced ASD risks.

## Figures and Tables

**Table 1:** Genetic variations found in ASD in recent researches.

Region	Dysregulated genes listed	Ref.
Dorsolateral prefrontal cortex	Reduction in mGluR5	[41]
Anterior cingulate gyrus, motor cortex, thalamus	Reduction in Metaxin 2 (MTX2), Light polypeptide (NEFL), Solute carrier family 25, member 27 (SLC25A27)	[123]
Inhibitory neurons in brain	Augmentation in GAD1, RELN, VIP, CHD7, PAX6, TBX1, CHD8, EHMT1, SATB2	[124]
Dorsolateral prefrontal cortex	Dysregulation of ErbB4, MMP2, NID1, TIMP1, COL4A3, RELN, ROBO1, ADORA2A, p21 (CDKN1A), 14- 3-3, HGF, FGFRL1, TSC1	[125]
